# Intrapleural alteplase and DNase for complex tuberculous pleurisy: a medical approach

**DOI:** 10.1002/rcr2.706

**Published:** 2021-01-03

**Authors:** Xiong Khee Cheong, Mohamed Faisal Abdul Hamid

**Affiliations:** ^1^ Respiratory Unit University Kebangsaan Malaysia Medical Centre Kuala Lumpur Malaysia

**Keywords:** Alteplase, DNase, intrapleural fibrinolysis, tuberculous pleurisy

## Abstract

Tuberculous pleurisy is extra‐pulmonary tuberculosis caused by *Mycobacterium tuberculosis* (MTB), which is one of the main cause of pleural effusions in developing countries. Intercostal chest catheter is useful for drainage of infected pleural fluid and facilitates sepsis control. However, management might be challenging in complex tuberculous pleural effusion as the septations within the effusion hinder pleural drainage. Intrapleural fibrinolysis therapy improved infected fluid drainage and septic parameter in parapneumonic effusions; however, there seems to be little data on its use in tuberculous pleurisy. In our case series of seven patients with complex tuberculous pleurisy, the use of intrapleural alteplase and deoxyribonuclease (DNase) facilitated fluid drainage which resulted in clinical and radiological improvement. These medications should not be confined to bacterial aetiology only as our case series highlights that in complex tuberculous pleurisy, intrapleural alteplase and DNase may be used as an adjunctive treatment which are proven to be successful and safe.

## Graphical abstract

In this case series, we demonstrated successful treatment of complex tuberculous pleural effusion with intrapleural alteplase and deoxyribonuclease (DNase) as adjunctive therapy with anti‐tuberculosis (TB) agents.

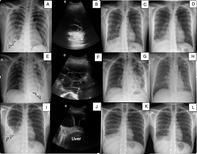

## Introduction

Tuberculosis (TB) is a communicable disease that causes morbidity and mortality globally. Tuberculous pleural effusion (TBE)/pleurisy is the second common extra‐pulmonary TB after lymphatic involvement. It is caused by the delayed hypersensitivity reaction in response to the rupture of subpleural *Mycobacterium tuberculosis* (MTB) caseous foci or reactivation of TB. Invasive procedures such as video‐assisted thoracoscopic surgery (VATS) or decortication are needed in poorly drained complex TBE, which is only limited to low operative risk patients. Intrapleural fibrinolysis has been used in the management of complex effusion; however, there are limited data on the management of tuberculous pleurisy. In this case series, we demonstrated successful treatment of complex TBE with intrapleural alteplase and deoxyribonuclease (DNase) as adjunctive therapy with anti‐TB agents. The demographic and clinical characteristics of the patients are displayed in Table 1.

**Table 1:** Demographic and clinical characteristics of patients.

Demographic data		Case 1	Case 2	Case 3	Case 4	Case 5	Case 6	Case 7
Age (years)		29	54	30	38	54	56	25
Sex		Male	Male	Male	Male	Male	Female	Female
Outcome		Remission	Remission	Remission	Remission	Remission	Remission	Remission
Comorbidities		None	Diabetes, hypertension	None	None	None	Diabetes, hypertension	None
Initial symptoms		Fever, cough,^*^ haemoptysis, weight loss^†^	Fever, cough^*^, weight loss^†^	Fever, cough^*^, weight loss^†^	Cough^*^, haemoptysis	Fever, cough,^*^ haemoptysis, weight loss^†^	Fever, cough^*^	Fever, cough,^*^ lymph node^‡^
Pleural fluid analysis								
Site of effusion		Right pleural	Left pleural	Right pleural	Right pleural	Right pleural	Right pleural	Left pleural
Macroscopic appearance of fluid		Serous	Purulent	Serous	Serous	Purulent	Serous	Serous
Pleural fluid LDH (U/L)		679	4914	1034	507	2907	439	579
Pleural fluid protein (g/L)		58	64	51	56	62	45	59
Glucose (mmol/L)		2.1	Not available	Not available	Not available	4.8	8.1	4
Lymphocytic effusion		Yes	Yes	Yes	Yes	Yes	Yes	No
Pleural fluid ADA (U/L)^§^		64.8	136.6	76	Not available	Not available	Not available	Not available
Pleural fluid MTB PCR		Negative	Negative	Not available	Negative	Negative	Positive	Not available
Pleural fluid MTB culture		No growth	No growth	No growth	No growth	No growth	Not growth	No growth
Pleural catheter								
Tube size		12 Fr Seldinger tube	12 Fr Seldinger tube	12 Fr Seldinger tube	8 Fr pigtail tube	12 Fr Seldinger tube	12 Fr Seldinger tube	8 Fr pigtail tube
Pleural drainage (mL/day)								
Day 0 (baseline)		10	0	110	50	0	0	0
Day 1		1100	500	1050	2200	340	500	350
Day 2		340	300	750	1050	310	600	150
Day 3		10	120	150	1090	100	100	550
Blood parameter								
Haemoglobin (g/dL)	Admission	12.6	10.6	10.9	14.8	11.2	10.8	10.8
	Discharge	12	11.1	12.7	14.1	13.6	10.1	9.3
White cell count (×10^9^)	Admission	6.6	11.5	7.0	6.0	3.9	5.5	8.1
	Discharge	7.8	10.5	3.7	7.0	5.7	8.1	7.4
C‐reactive protein (mg/dL)^¶^	Admission	17.3	7.8	18.9	19.8	13.6	22.4	9.1
	Discharge	5.0	2.5	12.1	13.8	14.7	5.3	5.6
Hospital stay from first dose alteplase/DNase (days)		5	4	4	7	4	8	8
Interval between anti‐TB and IPFT (days)		Day 4 anti‐TB	Day 50 anti‐TB	Three days prior to initiation of anti‐TB	Four days prior to initiation of anti‐TB	Day 7 anti‐TB	Day 6 anti‐TB	Day 10 anti‐TB

^*^ Duration of symptoms for more than two weeks.

^†^ Loss of weight more than 5 kg.

^‡^ Cervical lymphadenopathy.

^§^ ADA: normal range < 24 U/L.

^¶^ CRP: normal range < 0.5 mg/dL.

ADA, adenosine deaminase; CRP,C‐reactive protein; DNase, deoxyribonuclease; IPFT, intrapleural fibrinolytic therapy; LDH, lactate dehydrogenase; MTB, *Mycobacterium tuberculosis*; PCR, polymerase chain reaction; TB, tuberculosis.

## Case Series

### Case 1

**Figure 1:**
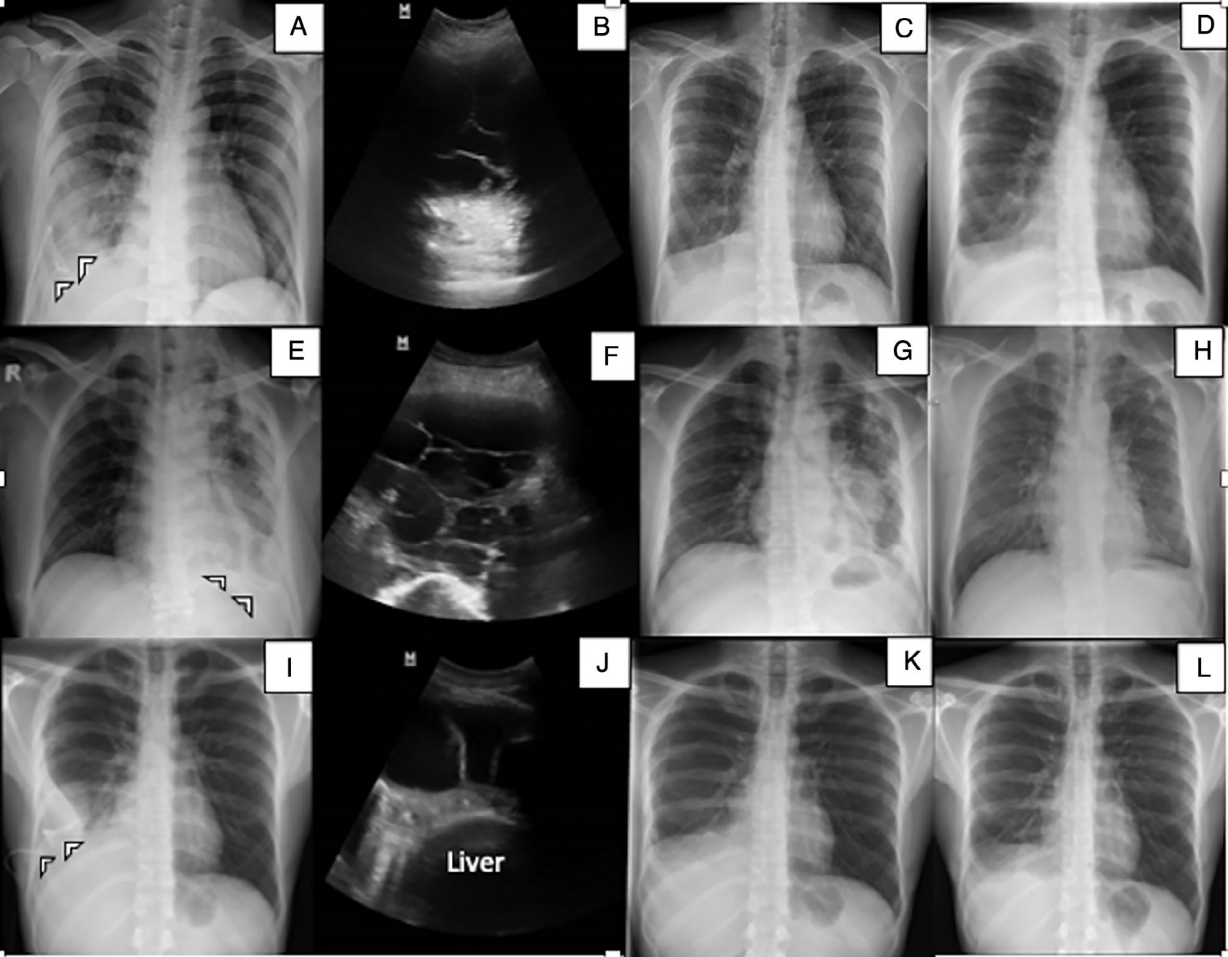
Chest radiographs on initial presentation, showing unilateral pleural effusion in case 1 (A), case 2 (E), and case 3(I); Intercostal chest catheter in situ (arrows). Ultrasound thorax revealed multiloculated effusion in case 1 (B), case 2 (F), and case 3 (J). Chest radiographs day 7 post intrapleural alteplase–deoxyribonuclease (DNase) and two months post treatment showed residual effusion and residual pleural thickening, respectively, in case 1 (C, D), case 2 (G, H) and case 3 (K, L).

A 29‐year‐old gentleman was admitted for partially treated pneumonia with right parapneumonic effusion. He had close contact with his colleague who was diagnosed with smear‐positive pulmonary TB (PTB). He required nasal prong 3 L/min oxygen due to hypoxaemia. Initial chest radiograph showed right moderate pleural effusion (Fig. 1A). He was treated empirically with intravenous ceftriaxone 2 g daily. Intercostal chest catheter (ICC) was inserted which only drained in the first three days due to septations (Fig 1B). Pleural fluid analysis was exudative. The diagnosis of TBE was confirmed by a lymphocyte‐filled pleural fluid with raised adenosine deaminase (ADA) of 64.8 U/L and positive Xpert MTB/rifampicin assay. Hence, fixed‐dose combination of anti‐TB agent, (akurit‐4) was started and the antibiotic was withheld. We decided for intrapleural fibrinolytic therapy (IPFT) and he received three doses of alteplase 16 mg and DNase 5 mg in 24 h. Following that, there was a remarkable improvement of pleural drainage (Table 1 and Fig 1C‐D). Patient's fever and breathlessness subsided with improved septic parameters. He was discharged well and completed six months of anti‐TB therapy.

### Case 2

A 54‐year‐old gentleman with diabetes mellitus and hypertension, and smear‐negative PTB on day 50 presented with left complex empyema and underwent intensive phase anti‐TB therapy. He required venturi mask of 40% fraction of inspired oxygen on admission. Chest radiograph and ultrasound revealed left moderate pleural effusion (Fig 1E‐F). He was treated with intravenous tazobactam/piperacillin 4.5 g thrice daily with anti‐TB agent. Pleural fluid analysis as per Table 1. IPFT was decided after two days of poor drainage from ICC; he received three doses of alteplase 16 mg and DNase 5 mg as per our protocol. Drainage of fluid increased to 920 mL following IPFT. Patient's fever and breathlessness resolved with the improvement of infective parameters. Chest radiograph showed improvement of the effusion (Fig 1G‐H). He was discharged with amoxycillin/clavulanate 625 mg thrice daily for four weeks and maintenance therapy of anti‐TB agent.

### Case 3

A 30‐year‐old construction worker presented with complex pleural effusion (Fig 1I) with prolonged cough and fever. Intravenous ceftriaxone 2 g daily was initiated. ICC was inserted and drained minimal serous fluid due to loculated effusion (Fig 1J). Bronchoscopy lavage and blood culture were sterile. The elevated ADA of 76 U/L and lymphocyte‐rich effusion with positive Mantoux test supported the diagnosis of TBE (Table 1). He was started on anti‐TB agent, (akurit‐4), and the antibiotics were withheld. Three doses of sequential 16 mg alteplase and 5 mg DNase were administered as per our protocol. Patient's fever subsided with an improvement of septic parameters. Serial imaging revealed resolution of effusion (Fig 1K‐L). He was discharged and completed six months of anti‐TB therapy.

### Case 4

**Figure 2:**
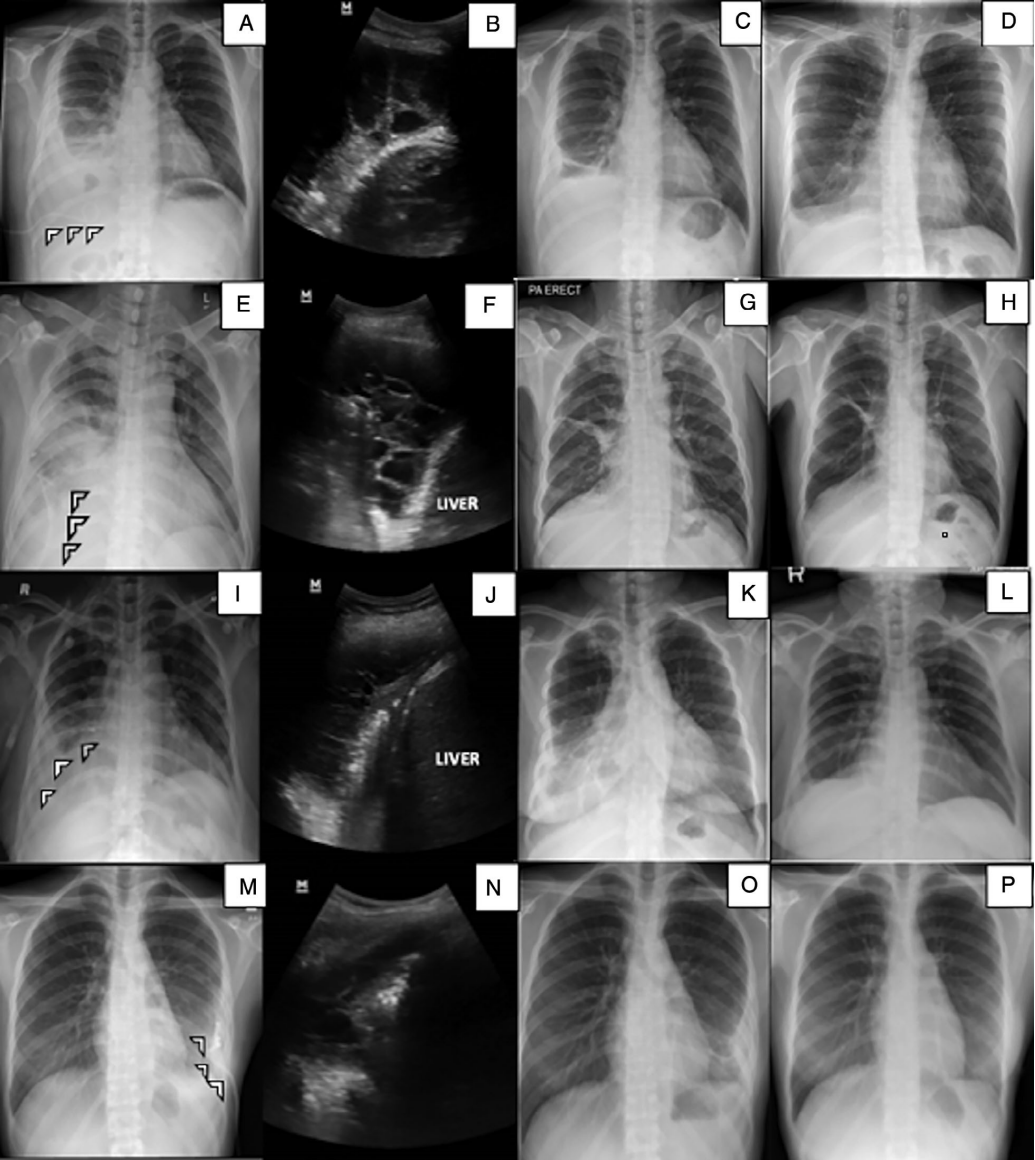
Chest radiographs on initial presentation, showing unilateral pleural effusion in case 4 (A), case 5 (E), case 6 (I), and case 7 (M); Intercostal chest catheter in situ (arrows). Ultrasound thorax revealed multiloculated effusion in case 4 (B), case 5 (F), case 6 (J), and case 7 (N). Chest radiographs day 7 post intrapleural alteplase–deoxyribonuclease (DNase) and two months post treatment showed residual effusion and residual pleural thickening, respectively, in case 4 (C, D), case 5 (G, H), case 6 (K, L), and case 7 (O, P).

A 39‐year‐old gentleman was referred for recurrent complex right pleural effusion (Fig 2A‐B). He had close contact history with his family who was treated for PTB. He received intravenous tazobactam/piperacillin 4.5 g thrice daily. Pleural fluid analysis as demonstrated in Table 1. Bronchoscopy lavage and culture were sterile. Computed tomography (CT) thorax revealed right pleural effusion with necrotic mediastinal and hilar lymphadenopathy. The diagnosis of tuberculous pleurisy was made based on lymphocytic filled effusion with positive Mantoux test. There was no lymph node biopsy or needle aspiration performed. We decided on IPFT due to persistent sepsis and poor drainage from ICC after 72 h of insertion. The patient improved clinically and radiologically following IPFT (Fig 2C‐D). He was discharged and completed six months of anti‐TB therapy.

### Case 5

A 54‐year‐old active smoker presented with complex right effusion (Fig 2E). CT thorax showed right empyema with thickened enhancing pleural. Pleural fluid analysis as per Table 1. ICC was inserted and drained purulent fluid. Intravenous tazobactam/piperacillin 4.5 g thrice daily was started empirically. He was then treated for tuberculous pleurisy based on lymphocytic effusion, positive Mantoux test, and history of close contact with PTB patient. Bronchoscopy lavage revealed a sterile culture. Anti‐TB therapy was initiated. IPFT was given as per protocol due to septated effusion (Fig 2F) with poor drainage. The patient's cough and fever resolved with improvement on fluid drainage. Serial imaging had remarkable improvement (Fig 2G‐H). He was discharged and completed six months of anti‐TB therapy.

### Case 6

A 56‐year‐old lady with diabetes mellitus and hypertension was admitted for complex right pleural effusion (Fig 2I‐J). Bronchoscopy lavage and blood culture were sterile. Pleural fluid analysis as described in Table 1. The diagnosis of tuberculous pleurisy was made based on lymphocytic effusion and positive pleural fluid MTB polymerase chain reaction (PCR) assay. Intravenous tazobactam/piperacillin was withheld and akurit‐4 was initiated. IPFT was given due to persistent sepsis and poor drainage following the insertion of ICC. There was an improvement on pleural drainage, radiological (Fig 2K‐L) and general condition following IPFT. She was discharged and completed six months of anti‐TB therapy.

### Case 7

A 25‐year‐old lady presented with complex left parapneumonic effusion (Fig 2M‐N) and cervical lymphadenopathy. Pleural fluid analysis as described in Table 1. There was no growth in blood and pleural fluid culture. Intravenous amoxicillin/clavulanic acid was initiated; however, anti‐TB agents were started following histopathology of cervical lymph node that revealed chronic granulomatous inflammation. We decided for IPFT after poor drainage of pleural fluid. Three doses of sequential 16 mg alteplase and 5 mg DNase were given as per our protocol. Following that, there was an improvement of pleural drainage (Table 1). Patient's general condition and cough improved and she remained afebrile. Serial imaging showed improvement (Fig 2O‐P). She was discharged well with anti‐TB therapy.

## Discussion

TB is a common cause of pleural effusion worldwide [1]. Tuberculous pleurisy is the second common extra‐pulmonary TB after lymphatic involvement [1]. We described seven patients with TBE who were successfully treated with anti‐TB agents and adjunctive intrapleural alteplase and DNase.

The diagnosis of tuberculous pleurisy is challenging due to the low pleural bacterial yield from pleural fluid AFB smear (less than 5% positivity) and MTB culture (24–58% sensitivity) [2, 3]. Pleural biopsy histology and culture via closed needle biopsy or medical thoracoscopy remain the gold standard methods (93% sensitivity) [2, 3]. ADA assay has high accuracy for the diagnosis of TB pleurisy (92% sensitivity and 90% specificity) [4]. The diagnosis of TBE in our cohort was based on the clinical and pleural fluid analysis. Two patients had positive bronchoalveolar lavage for gene Xpert and concomitant raised ADA of more than 40 U/L, one patient had a positive lymph node biopsy, and one patient had a positive pleural fluid MTB PCR.

The World Health Organization (WHO) and American Thoracic Society (ATS) recommend six months of anti‐TB regimen for the treatment of pleural TB which is similar to PTB [5, 6]. Therapeutic thoracentesis as adjunctive therapy to anti‐TB in the management of tuberculous pleurisy remains controversial; it should be considered in patients with symptomatic large effusions [7, 8]. Surgical decortication is indicated in persistent sepsis with poorly drained complex effusions depending on the patient's fitness. The timing of surgery remains debatable among pulmonologists and thoracic surgeons for TBE. Generally accepted timing of surgery is after at least two months of anti‐TB therapy to achieve optimal bacterial suppression prior to operation [9]. However, delay in surgical intervention might lead to patient's clinical deterioration and increases the perioperative mortality.

Recently, a minimally invasive approach with IPFT had demonstrated good outcomes following Multi‐Center Intrapleural Sepsis Trial (MIST‐2); 95% of patients showed improvement on pleural drainage and reduction in hospital stay without requiring surgical intervention [10]. Unfortunately, tuberculous‐related effusion was only in the placebo arm in MIST‐2 trial.

Few studies reported on the use of intrapleural urokinase or streptokinase in tuberculous effusion to facilitate pleural fluid drainage and the occurrence of residual pleural thickening [8, 11, 12]. There is limited literature on the use of intrapleural alteplase (with supplementary DNase) in complex tuberculous pleurisy [13, 14]. Our cases showed that intrapleural alteplase and DNase facilitated more infected fluid drainage with clinical resolution of fever and dyspnoea. Its potential for the reduction of residual pleural thickening still requires large cohort studies in the future. There is no optimal dose on alteplase in intrapleural use. We administered three doses of 16 mg alteplase (with supplementary DNase 5 mg) to simplify the pharmacy dispensary as in our formulary, one ampoule of alteplase contains 50 mg and it should be utilized within 24 h after reconstitution. There were no pleural or systemic bleeding events observed in our patients with stable haemoglobin level which showed the safety of this modified regimen of intrapleural alteplase–DNase. Larger studies could be conducted in the future to evaluate the efficacy of intrapleural alteplase–DNase in complex TB pleurisy as an alternative therapy to surgical intervention.

### Disclosure Statement

Appropriate written informed consent was obtained for publication of this case series and accompanying images.
